# Cooperation Between Systemic IgG1 and Mucosal Dimeric IgA2 Monoclonal Anti-HIV Env Antibodies: Passive Immunization Protects Indian Rhesus Macaques Against Mucosal SHIV Challenges

**DOI:** 10.3389/fimmu.2021.705592

**Published:** 2021-08-03

**Authors:** Siqi Gong, Samir K. Lakhashe, Dinesh Hariraju, Hanna Scinto, Antonio Lanzavecchia, Elisabetta Cameroni, Davide Corti, Sarah J. Ratcliffe, Kenneth A. Rogers, Peng Xiao, Jane Fontenot, François Villinger, Ruth M. Ruprecht

**Affiliations:** ^1^Texas Biomedical Research Institute, San Antonio, TX, United States; ^2^New Iberia Research Center, University of Louisiana at Lafayette, Lafayette, LA, United States; ^3^Department of Microbiology, Immunology, and Molecular Genetics, University of Texas Health San Antonio, San Antonio, TX, United States; ^4^Institute for Research in Biomedicine, Bellinzona, Switzerland; ^5^Humabs BioMed, A Subsidiary of Vir Biotechnology, Bellinzona, Switzerland; ^6^University of Pennsylvania, Philadelphia, PA, United States; ^7^Department of Biology, University of Louisiana at Lafayette, Lafayette, LA, United States

**Keywords:** dimeric IgA, IgG, passive mucosal and systemic immunization, rhesus macaque model, SHIV, immune exclusion

## Abstract

Understanding the interplay between systemic and mucosal anti-HIV antibodies can provide important insights to develop new prevention strategies. We used passive immunization *via* systemic and/or mucosal routes to establish cause-and-effect between well-characterized monoclonal antibodies and protection against intrarectal (i.r.) SHIV challenge. In a pilot study, for which we re-used animals previously exposed to SHIV but completely protected from viremia by different classes of anti-HIV neutralizing monoclonal antibodies (mAbs), we made a surprise finding: low-dose intravenous (i.v.) HGN194-IgG1, a human neutralizing mAb against the conserved V3-loop crown, was ineffective when given alone but protected 100% of animals when combined with i.r. applied HGN194-dIgA2 that by itself had only protected 17% of the animals. Here we sought to confirm the unexpected synergy between systemically administered IgG1 and mucosally applied dIgA HGN194 forms using six groups of naïve macaques (n=6/group). Animals received i.v. HGN194-IgG1 alone or combined with i.r.-administered dIgA forms; controls remained untreated. HGN194-IgG1 i.v. doses were given 24 hours before – and all i.r. dIgA doses 30 min before – i.r. exposure to a single high-dose of SHIV-1157ipEL-p. All controls became viremic. Among passively immunized animals, the combination of IgG1+dIgA2 again protected 100% of the animals. In contrast, single-agent i.v. IgG1 protected only one of six animals (17%) – consistent with our pilot data. IgG1 combined with dIgA1 or dIgA1+dIgA2 protected 83% (5/6) of the animals. The dIgA1+dIgA2 combination without the systemically administered dose of IgG1 protected 67% (4/6) of the macaques. We conclude that combining suboptimal antibody defenses at systemic and mucosal levels can yield synergy and completely prevent virus acquisition.

## Introduction

Worldwide, most new HIV-1 infections occur through mucosal exposures, including sexual transmission as well as perinatally acquired infections. The overwhelming majority of newly infected individuals harbor initially CCR5-tropic (R5) strains. As such, mucosal fluids and epithelial barriers represent portals of entry for HIV-1 for more than 90% of newly acquired infections. Mobilizing host immune defenses through vaccine strategies that include induction of mucosal immunity is clearly important.

Mucosal fluids contain different classes of immunoglobulins: IgM, IgG, and IgA. Depending on the mucosal fluid, either IgG or IgA predominate [reviewed in (1)]. While IgG can be synthesized by the subepithelial plasma cells, it also originates from the systemic circulation after crossing blood vessels, tissue dissemination, and transepithelial transport by the Fc neonatal receptor (FcRn). IgM and IgA destined for mucosal fluids are generated by subepithelial plasma cells as polymers. IgM is predominantly a pentamer that binds to the polymeric Ig receptor (pIgR) through the Joining (J) chain (2). IgA is produced by subepithelial plasma cells as dimer - also incorporating the J chain, which permits the transepithelial transport of dimeric IgAs (dIgAs). At the luminal site, pIgR undergoes proteolytic cleavage, leaving behind the secretory component (SC) that remains with IgM and dIgA to form secretory IgM (SIgM) and secretory IgA (SIgA), respectively (3). The human body generates more IgA per day than all other classes of immunoglobulins combined (4); most of this IgA is destined for transepithelial transport and entry into mucosal fluids. As such, SIgA needs to be replaced on an ongoing basis.

**Figure 1:**
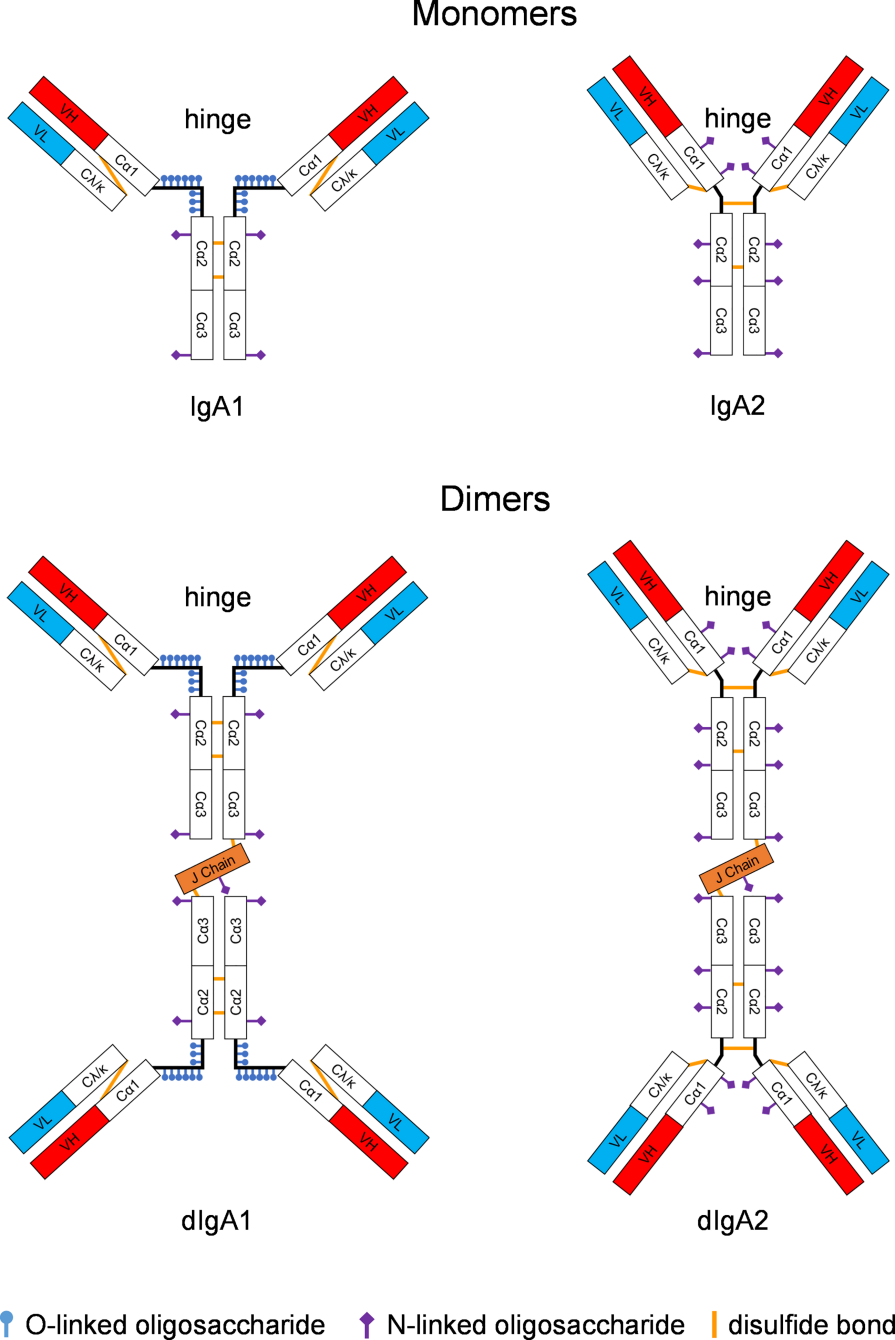
Structure of IgA monomers and dimers. IgA1 and IgA2 differ significantly in the hinge region. For IgA1 forms, the hinge is wide open and contains O-linked glycosylation sites. In contrast, the IgA2 molecule is more Y-shaped thus resembling the classical structure of IgG; the IgA2 hinge has some N-linked glycosylation sites. IgA isotypes vary widely in different animal species; only humans and some of the great apes have IgA1 versions with the wide-open hinge. Rhesus monkeys only have the IgA2-like form. Monomeric IgA molecules can be linked with the joining (J) chain to form dimers. Constant regions of the heavy chain are designated Cα1, Cα2, or Cα3. The light chain carries one constant region, Cκ or Cλ, respectively. The heavy and light chains are linked through disulfide bonds (ochre lines). The antigen combining site consists of the variable heavy (VH, red boxes) and the variable light (VL, turquois boxes) fragments.

In humans, IgA exists as two isotypes, IgA1 and IgA2 (5). In the systemic circulation, most IgA is present in monomeric form (**Figure 1**). In mucosal fluids, dimers predominate as SIgAs. IgA1 and IgA2 differ mostly in the hinge region, which is significantly longer and wide-open in IgA1 compared to that present in IgA2. As a result, IgA1 is more like a T-shaped molecule (6), whereas IgA2 resembles the classical Y-shape of IgG (7). The hinge of IgA1 also contains multiple O-linked glycosylation sites that are completely absent in the IgA2 hinge, which instead has a few N-linked glycosylation sites (8–10). Overall, IgA1 molecules are more flexible than IgA2.

Are mucosal antibodies of the different Ig classes protective against mucosal HIV transmission? To answer this question, we have used simian-human immunodeficiency viruses (SHIVs), chimeras that express HIV-1 envelope in an SIV backbone; SHIVs have been adapted to be replication competent and pathogenic in rhesus macaques (RMs). We have generated recombinant monoclonal antibodies (mAbs) with identical epitope specificity but different Ig backbones, including IgM, dimeric IgA1 (dIgA1), dimeric IgA2 (dIgA2), and IgG1. To test whether these recombinant mAbs could provide protection in the mucosal lumina, we applied them topically 30 min before mucosal SHIV challenge. Our passive mucosal immunization showed significant protection for IgM (11), dIgA (12), and IgG (13) mAbs. These studies gave proof-of-concept that mucosal antibodies can prevent SHIV transmission.

Next, our group sought to examine the interaction between monoclonal dIgA2 and IgG forms in the mucosal compartment. We were prompted to do this based upon data from the RV144 Phase 3 vaccine efficacy study (14), where the protective principle was non-neutralizing IgG with vector function predominantly ADCC (15). Remarkably, IgA directed against HIV envelope interfered with the protective role of systemic IgG. We wondered whether this would be the case also in mucosal fluids. We performed a pilot study in RMs that had a history of exposure to live virus under the protective umbrella of passively administered mAbs given either through the intravenous (i.v.) or intrarectal (i.r.) routes. None of these animals had ever been viremic, and they had no residual human mAbs or anti-human antibody responses (16). To mimic the distribution of IgG in the systemic compartment throughout body tissues as well as mucosal fluids, we administered a suboptimal dose of a human neutralizing anti-HIV Env IgG mAb, HGN194-IgG1 (17). This mAb targets the conserved V3 loop crown. The intravenous passive immunization was performed 24 hours before i.r. SHIV challenge. The dIgA2 form of the same mAb was administered i.r. 30 min before the SHIV challenge. By itself, the dIgA2 form had only protected 17% of RMs given *via* passive mucosal immunization (12). The pilot study performed in the SHIV-exposed, aviremic RMs yielded a surprise finding (16): complete protection of all animals given low-dose i.v. HGN194-IgG1 together with i.r. applied dIgA2. In contrast, HGN194-IgG1 as single-agent at the low dose given protected none of the treated animals (16). This finding was so unexpected and of such potential importance that this current study’s aim was to reproduce these findings in naïve animals.

Here we were able to achieve 100% protection of naïve RMs given the suboptimal dose of i.v. HGN194-IgG1 combined with the minimally protective i.r. HGN194-dIgA2. These data indicate that our initial observation in SHIV-exposed RMs is reproducible.

## Materials and Methods

### Virus

The SHIV-1157ipEL-p (18) stock was grown in RM peripheral blood mononuclear cells (PBMC); it had a p27 concentration of 792 ng/ml and 7.8 x 10^5^ 50% tissue culture infectious doses (TCID_50_)/ml as measured in TZM-bl cells.

### Antibody Production, and Quality Control

The mAbs were produced from the same source materials and had the same general properties as described (12). Briefly, recombinant mAbs were expressed in Expi293 cells (Gibco) cultured at 37°C and 8% CO2 in Expi293 expression medium (Gibco). Cells were co-transfected with plasmids encoding heavy (HGN194-IgG1, HGN194-IgA1, or HGN194-IgA2) and light chains (HGN194-IgL). In addition, a J chain expression plasmid was used to produce dimeric IgA forms. Transfections were performed using PEI MAX 40 kDa (Polysciences – Brunschwig) as a transfection agent at a PEI : DNA ratio of 3:1 and 1 µg of total DNA/ml cell culture. Transfected cells were supplemented three days after transfection with Soy hydrolysate (Sigma) and glucose (Sigma) and cultured for an additional four days. Cell culture supernatants were collected seven days after transfection and pre-clarified by 20 min centrifugation at 6,000 rpm and subsequently loaded with a peristaltic pump on a Pall AcroPak 500 cm^2^ 0.8/0.2 µm filter capsule (VWR) previously equilibrated with phosphate-buffered saline (PBS).

Recombinant IgG1 antibodies were affinity purified on an ÄKTAxpress FPLC device using 5 ml HiTrap Mab Select Xtra columns followed by buffer exchange to PBS using HiPrep 26/10 desalting columns. Recombinant IgA forms were purified either on 5 ml CaptureSelect™ IgA followed by buffer exchange to PBS using HiPrep 26/10 desalting columns. The final products were sterilized by filtration through 0.22 µm filters and supplemented with 0.02% polysorbate 80.

The purified mAbs were quantified using the BCA method according to the manufacturer’s instructions (Pierce). The binding of the recombinant mAbs to the HIV antigen UG37gp140 was assessed by ELISA. To determine the monomeric/multimeric state of the recombinant mAbs and to confirm the correct formation of IgA dimer, the purified mAbs were analyzed by SEC-UHPLC on an ACQUITY UPLC BEH200 SEC, 1.7 µm Column using a 1260 Infinity Quaternary Bio-inert LC instrument (Agilent). Purity was assessed by separation of the purified mAbs on SDS-PAGE under reducing as well as non-reducing conditions. Endotoxin levels were assessed with the Endosafe Nexgen-PTS/L.A.L. Endosafe-PTS cartridges method (Charles River). The neutralization assays against the challenge virus, SHIV-1157ipEL-p (18), were performed in TZM-bl cells as described (19) (**Figure S1**). When mAb combinations were tested, the concentration was the sum of the two mAbs mixed at an equal molar ratio.

### Animals

We enrolled 36 naïve, male, Indian-origin RMs (*Macaca mulatta*) between 2-4 years of age for this study. The RMs were bred and housed at the New Iberia Research Center (NIRC, New Iberia, LA, USA), University of Louisiana at Lafayette (UL Lafayette), in accordance with the recommendations in the Guide for the Care and Use of Laboratory Animals of USA. NIRC is an Association for Assessment and Accreditation of Laboratory Animal Care International-accredited facility. All procedures were approved by the Animal Care and Use Committee of the UL Lafayette.

All RMs were negative for Mamu B*08 and Mamu B*17 alleles. The PBMC of all RMs were isolated prior to the experiments and tested for their ability to support the replication of challenge virus, SHIV-1157ipEL-p (18). The p27 levels in the culture supernatants were measured using the SIV p27 Antigen Capture Assay kit (ABL Inc.). The RMs were randomized into six groups of six by age, bodyweight, Mamu A*01 status, TRIM5α/CD16/CD64 genotypes, and peak p27 levels produced by *in vitro* infected PBMC (**Table 1**).

**Table 1:** Group assignment of RMs.

Group	Animal ID	Age (year) Prestudy	Body weight (kg) Prestudy	Peak p27 (ng/ml)	MHC Typing			TRIM5α	CD16			CD64 alleles					
					A*01	B*08	B*17	Restriction	3A-1	3A-2	3A-3	1	2	3	4	6	7
1	A15X086	1.42	2.25	38	+	–	–	High^1^	x	x		xx					
	A15X022	1.87	2.60	138	+	–	–	Susceptible^2^		xx		xx					
	A15X046	1.76	3.65	164	+	–	–	Moderate^3^	xx			xx					
	A15X033	1.82	2.95	168	–	–	–	Moderate	x	x		xx					
	A15T004	1.92	3.20	329	–	–	–	High		xx		xx					
	A15T006	1.90	2.75	500	–	–	–	High	x	x		Data not available					
2	A15X039	1.79	3.20	16	–	–	–	High		xx		xx					
	A15X024	1.84	2.35	30	+	–	–	Moderate	x	x		xx					
	A15X012	1.89	3.15	348	–	–	–	Moderate	xx			xx					
	A15X019	1.87	3.95	358	–	–	–	High	xx			xx					
	A15T001	1.95	2.45	890	–	–	–	Moderate	x	x							xx
	A15X053	1.71	2.55	912	+	–	–	High	x		x		x				x
3	A15X085	1.44	2.85	24	–	–	–	Moderate	x	x		xx					
	A15X021	1.87	3.10	58	–	–	–	High		xx		xx					
	A15X089	1.36	2.45	351	+	–	–	High		xx		x					x
	A14T016	2.66	4.05	378	–	–	–	High		xx		Data not available					
	A15X076	1.58	3.05	565	+	–	–	Susceptible	xx			xx					
	A14T012	2.76	4.25	868	+	–	–	Moderate	x	x		xx					
4	A15X088	1.38	2.70	46	–	–	–	Moderate		xx		xx					
	A15T013	1.72	2.65	168	–	–	–	Moderate		xx		xx					
	A15X017	1.87	2.85	268	+	–	–	High	x	x		xx					
	A15X040	1.79	4.15	293	–	–	–	Moderate		xx		x				x	
	A15T005	1.91	3.10	550	+	–	–	High	x	x			xx				
	A14T005	2.83	4.30	671	–	–	–	High	xx			x	x				
5	A15X065	1.64	3.00	36	–	–	–	High	x		x	Data not available					
	A15X066	1.63	3.05	74	+	–	–	High	x	x		x				x	
	A15X082	1.50	2.60	132	–	–	–	High		xx		Data not available					
	A15X090	1.35	2.50	333	–	–	–	Moderate	x	x		x					x
	A15X020	1.87	3.10	521	+	–	–	Moderate	x		x	xx					
	A14T015	2.72	3.95	838	+	–	–	High	x	x		xx					
6	A15X062	1.65	2.30	39	+	–	–	High	x	x		xx					
	A15X069	1.62	2.85	199	–	–	–	Susceptible		xx				xx			
	A15X056	1.70	2.90	263	+	–	–	Moderate	xx			x				x	
	A15T002	1.93	2.60	295	–	–	–	High	x	x		xx					
	A15T003	1.93	3.45	395	–	–	–	Moderate		xx		x	x				
	A14T001	2.92	4.30	467	–	–	–	High	xx			x					x

^1^High = TFP and or CypA.

^2^Susceptible = Q allele.

^3^Moderate = heterozygous with one Q allele.

### Passive Immunization and Mucosal SHIV-1157ipEL-p Challenge

**Figure 2:**
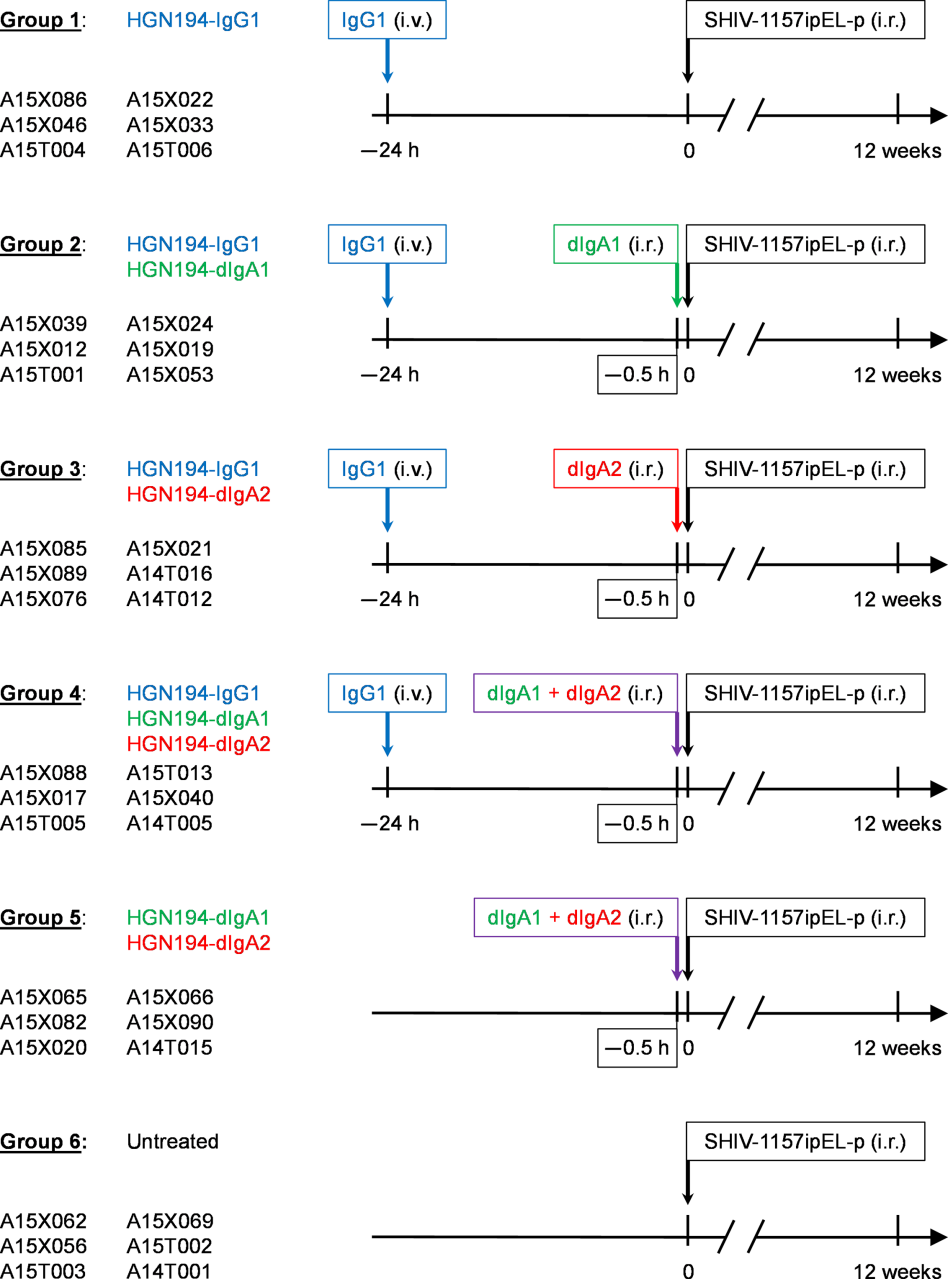
Study design and timeline for the passive immunizations with different forms of the human monoclonal antibody (mAb) HGN194 (17). This human mAb recognizes the conserved crown of the V3 loop. HGN194-IgG1 (blue) was administered intravenously, whereas the dimeric IgA (dIgA) forms were given intrarectally (i.r.) (dIgA1, green; dIgA2, red). At time 0, all animals were challenged with a single high dose of the tier 1, R5, clade C SHIV-1157ipEL-p (18) through the i.r. route. Viral RNA (vRNA) loads were assessed prospectively for a period of 12 weeks. Control animals (Group 6) were left untreated and underwent SHIV challenge at time 0.

The 36 RMs were treated according to the experimental timeline (**Figure 2**); IgG1 mAbs were administered i.v. at a dose of 1.45 mg/kg 24 h before viral challenge, and dIgA mAbs were given intrarectally i.r. each at a dose of 1.25 mg/RM (in 2.1 ml PBS at a final concentration of 0.595 mg/ml) 30 min before the viral challenge, respectively. All animals were challenged i.r. with 31.5 50% animal infectious doses (AID_50_) (equivalent to 1.7 x 10^5^ TCID_50_) of the R5 clade C SHIV-1157ipEL-p (18).

### Plasma Viral RNA Levels

Plasma samples were collected on the day of the challenge and thereafter for 12 weeks. RNA was isolated from the plasma using QIAamp Viral RNA Mini Kits (Qiagen), and viral RNA (vRNA) levels were measured by quantitative reverse-transcriptase polymerase chain reaction (qRT-PCR) for SIV *gag* sequences. The sensitivity of the assay was 100 copies/ml.

### Statistical Analyses

Kaplan-Meier analyses and log-rank tests were performed using GraphPad Prism version 9.1.0.221 for Windows (GraphPad software LLC).

## Results

### A Minimally Protective Dose of HGN194-IgG1 Given i.v., Combined With Weakly Protective HGN194-dIgA2, Prevented Viremia After High-Dose SHIV-C Challenge

**Figure 3:**
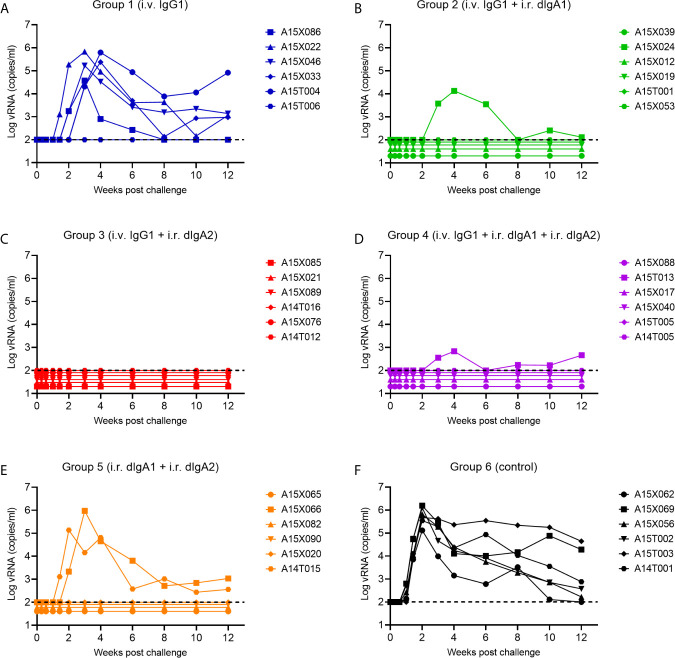
Viral RNA (vRNA) loads of experimental Groups 1 through 6 following a single high-dose intrarectal (i.r.) challenge with the tier 1, R5 clade C SHIV-1157ipEL-p (18). **(A–E)**, Groups 1 through 5 were passively immunized with different classes of the human mAb HGN194. The IgG1 form was given i.v., whereas the dIgA1 and/or dIgA2 forms were administered i.r. The untreated Group 6 **(F)** served as control. For details, please see the timeline in **Figure 2**.

In SHIV-exposed but uninfected animals, we had observed a strong synergy between two forms of HGN-194: IgG1 (given i.v.) and dIgA2 (given i.r.) (16). We sought to replicate these findings in naïve RMs. Group 1 RMs received i.v. HGN194-IgG1 only at a low dose of 1.45 mg/kg. Five out of six RMs became viremic within three weeks post-challenge (**Figure 3A**). All untreated controls became viremic and had high peak viral RNA (vRNA) loads (**Figure 3F**). This indicates that low-dose HGN194-IgG1 given systematically alone provided minimal protection against the SHIV-C challenge.

**Figure 4:**
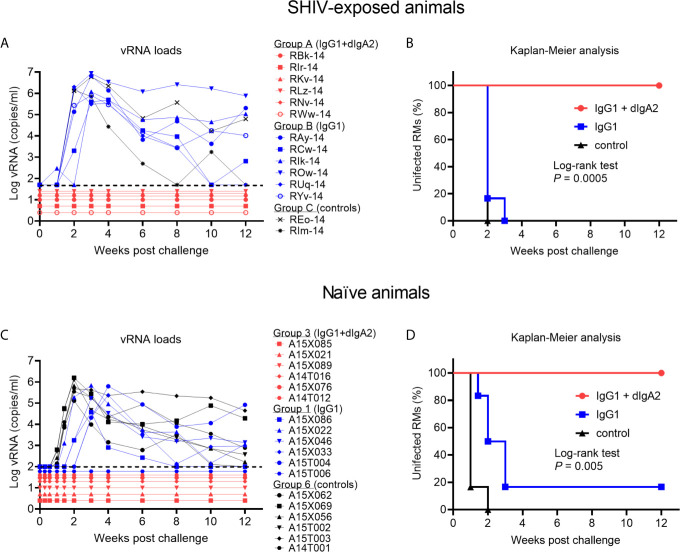
Complete protection by the combination of IgG1 + dimeric IgA2 (dIgA2) forms of the human mAb HGN194 seen in SHIV-exposed animals is reproducible in naïve macaques. Data performed earlier in SHIV-exposed, but never viremic rhesus macaques are shown in panels **(A, B)**; ^*^the data have been published in Sholukh et al., 2015 (16). Indian-origin rhesus macaques (RMs) in Group B were given the IgG1 form of HGN194 (IgG1; blue symbols) at the low dose of 1.45 mg/kg 24 h before the single high dose intrarectal (i.r.) SHIV challenge. Control animals (Group C) were left untreated (black symbols). All animals of Groups B and C became highly viremic. In contrast, none of the animals in Group A given the same low dose IgG1 as the animals in Group B together with dIgA2 administered topically had become viremic in the study by Sholukh et al. (16). **(C)** vRNA loads of Groups 3, 1, and 6 of the current study treated identically as those in panel **(A)**. Animals depicted in panel **(C)** were naïve at enrollment. vRNA loads are almost identical to those in panel **(A)**, with the exception that one of the six animals given i.v. IgG1 remained aviremic (blue symbol RM A15T006). Panels **(B, D)**, Kaplan-Meier analysis of the vRNA load data presented in **(A)** or **(C)**, respectively. Both experiments showed 100% protection against viremia throughout the 12 weeks of follow up [red line, **(B, D)**]. Panels **(A, B)**, adapted from Sholukh et al. (16) with permission.

However, when the suboptimal low-dose i.v. HGN194-IgG1 was combined with i.r. HGN194-dIgA2 (12) for the RMs in Group 3, all animals resisted the challenge and remained completely aviremic throughout (**Figure 3C**). The-time-to-viremia was compared using the log-rank test. Clearly, the combination of i.v. IgG1 and i.r. dIgA2 provided significant protection against the single high-dose SHIV-C challenge (Group 3 *vs.* Control Group 6, P = 0.0005), and the protection provided by the combination was significantly better than i.v. IgG1 alone (P =0.005) (**Figure 4** **)**. Since passive immunization with i.r. HGN194-dIgA2 alone had protected only one out of six naïve RMs in the past (12), the strong protection of the combination seen in the current study suggests synergy between systemically applied HGN194-IgG1 and topically administered HGN194-dIgA2. The combination of these two antibodies given by different routes significantly improved the protection provided by each individual treatment. This result in naïve animals confirmed our earlier finding in the SHIV-exposed but uninfected animals (16).

### Adding HGN194-IgG1 at a Suboptimal i.v. Dose to HGN194-dIgA1 Given i.r. Did Not Provide More Protection

Next, we sought to examine whether the strong synergy observed between i.v. HGN-194-IgG1 and i.r. HGN-194-dIgA2 would boost the strong protection provided by HGN194-dIgA1 given i.r as single agent. Earlier, i.r. HGN194-dIgA1 had protected five out of six RMs (86%; Watkins 2013). Group 2 RMs received HGN194-IgG1 i.v. at –24 h followed by HGN194-dIgA1 given i.r. at –30 min before SHIV-C challenge. Five out of six RMs were protected from viremia (**Figure 3B**). The combination of i.v. IgG1 and i.r. dIgA1 afforded significant protection compared to the controls (time-to-viremia, P = 0.0005). However, the degree of protection against the same SHIV-C challenge was identical to that seen earlier with single-agent topical HGN194-dIgA1 (12), suggesting that the addition of systemic low-dose HGN194-IgG1 did not provide additional benefit.

### Combining i.r. HGN194-dIgA1 With i.r. dIgA2 Yielded a Similar Level of Protection Compared to HGN194-dIgA1 Given i.r. as Single Agent

Next, we examined the interaction between HGN194-dIgA1 and HGN194-dIgA2 – both given topically. We treated RMs in Group 5 with a combination of full-dose HGN194-dIgA1 (1.25 mg/RM i.r.) and full-dose HGN194-dIgA2 (1.25 mg/RM i.r.) in a total of 2.1 ml PBS. Upon high-dose SHIV-C challenge by the rectal route, only two out of six RMs became viremic (**Figure 3E**), indicating significant protection compared to controls (time-to-viremia, P = 0.0046). However, the full-dose HGN194-dIgA1 given i.r. by itself had protected five out of six RMs against the same SHIV-C challenge (12), suggesting that adding full-dose HGN194-dIgA2 to dIgA1 did not provide more protection.

### Low-Dose Systemic HGN194-IgG1, Combined With Topical HGN194-dIgA1 + dIgA2, Provided Incomplete Protection Against High-Dose i.r. SHIV-C Challenge

Finally, we tested the triple combination of systemic low-dose HGN194-IgG1 given at –24 h followed by HGN194-dIgA1 + HGN194-dIgA2 given i.r. at –30 min before SHIV-C challenge. When challenged with a single high-dose SHIV-C, five out of six RMs never became viremic (**Figure 3D**), indicating significant protection was provided by the combination compared to controls (time-to-viremia, P = 0.0005). The RM with breakthrough infection had a relatively low peak vRNA load (682 copies/ml) compared to an average of 7.4 x 10^5^ copies/ml in the control group. Nevertheless, the triple mAb combination did not provide complete sterile protection.

## Discussion

Here we showed i) a striking 100% protection by combining a minimally protective dose of i.v. HGN194-IgG1 with mucosally applied, minimally protective HGN194-dIgA2 – this time in naïve macaques. In other words, our data generated earlier (16) in SHIV-exposed but aviremic RMs were reproducible in naïve animals; and ii) virus-exposed but aviremic animals can be enrolled in subsequent studies while yielding expected outcomes. This aspect has importance for the current severe problems with enrolling RMs of Indian-origin given the imbalance of supply and demand.

We also sought to examine the interactions between different classes/subtypes of mAb HGN194 beyond only i.v. IgG1 and i.r. dIgA2. The extended data revealed no additional benefit by adding a sub-protective dose of HGN194-IgG1 (given i.v.) or HGN194-dIgA2 (applied topically) to the highly protective HGN194-dIgA1 given by the i.r. route. In our earlier study (12), dIgA1 topically administered as single agent had protected 83% of the animals. In current study, no experimental group treated with topical dIgA1 in combination with other forms of HGN194 exceeded this degree of protection. Due to animal resource constraints, we could not repeat the single-agent topical administration of dIgA1 or dIgA2 (12) in the current study.

Of note, all different forms of HGN194 were built using human mAb backbones. There is strong homology between IgG1 and dIgA2 with the corresponding rhesus monkey immunoglobulins. However, RMs only have the Y-shaped IgA version that resembles IgA2 of humans and produce only IgA2-like antibodies [reviewed in (20)]. Only humans and some great apes have evolved to have the IgA1 forms with their wide-open hinges that contain a number of O-linked glycosylation sites. Although rhesus CD89 has been shown to bind both human IgA1 and IgA2 (21), how human IgAs interact with and activate rhesus CD89, or other Fc receptors remains unclear. The human dIgA2 may be able to activate certain immunological pathways that human dIgA1 cannot and assist the low-dose systemic IgG1 to protect the RMs from SHIV challenge completely. Nevertheless, our result showed that mucosal dIgA antibody responses play an important role in protecting against HIV infection. One potential mechanism is immune exclusion (22). The dIgAs in the mucosal lumen reduce or block the virus from passing through the epithelial layer, effectively reducing the challenge dose. Then, the IgG diffused from the circulation will have a high chance of preventing the low-level residual virus that crosses into the tissue from establishing infection or spreading systemically. If a vaccine could induce even weakly protective mucosal dIgA and systemic IgG responses together, there may be a chance for such a vaccine to provide sufficient protection to prevent HIV infection. *In vitro* neutralization assays with combinations of IgG1 and dIgA1 or dIgA2 revealed no synergy (**Figure S1**). Given that the HGN194 mAb isoforms have identical epitope specificity, this result for the mAb combinations tested was not surprising. Of note, it does not explain the strong synergy we observed reproducibly *in vivo*. The mucosal environment of live primates with its specialized anatomical structures, epithelial barriers, mucus proteins, and mucosal secretions will influence the interaction with incoming virus in ways that the TZM-bl assay cannot predict. To study the underlying mechanism(s) for the *in-vivo* mAb synergy, we are collaborating with Taylor et al. (23) using non-invasive imaging on live animals with positron emission tomography/computerized tomography (PET/CT) scanning.

Regarding result ii), the remarkable synergy between systemically administered IgG1 and topically applied dIgA2 forms of the same mAb we have discovered in SHIV-exposed RMs was confirmed in naïve animals. This suggests that virus-exposed but persistently aviremic and seronegative RMs could be recycled and used for new passive immunization studies in the future. Of note, we had ruled out anti-drug antibody (ADA) responses in the “recycled” RMs used in our earlier study (16) before re-enrollment for an additional passive immunization study. Reusing precious primates is especially important during the recent shortage of naïve RMs due to the COVID-19 pandemic. In fact, we argue that active vaccine studies can also be considered in “recycled” RMs with a history of prior SHIV exposure. To do this, it will be important to examine cell-mediated immunity (CMI) against viral antigens. We have done this before using the animals described in the study by Sholukh et al. (16). All of them had proliferative responses against SIV Gag in the CD4^+^, CD8^+^, or both CD4^+^ and CD8^+^ T-cell populations after *in vitro* stimulation with SIV Gag. We found that this assay was the most sensitive to reveal antigen-specific T-cell responses after exposure to live virus (16). In fact, the argument can be made that using RMs with a history of live-virus exposure in vaccine efficacy study is biologically relevant. Most individuals at risk for HIV acquisition through sexual interaction may have had prior exposure to live HIV without becoming infected. The estimated per-sexual intercourse probability of acquiring HIV from an infected source person ranges from 4 for insertive penile-vaginal intercourse to 138 for receptive anal intercourse per 10,000 exposures (24). Using consistently aviremic RMs with a history of exposure to live virus and Gag-specific CMI responses is reminiscent of the highly exposed, persistently seronegative sex workers who also had T-cell reactivity to HIV (25). Thus, one could argue that using virus-exposed but uninfected RMs may be more biologically relevant to real-life situations than using naïve RMs.

In summary, we demonstrated that the remarkable synergy between systemic IgG and mucosal IgA is real, using passive immunization as a rigorous test to show cause-and-effect for the protective role of mucosal antibodies in combination with IgG – data that have significance for *active vaccine strategies.* To date, mucosal/systemic IgG and mucosal IgA have been generated by HIV gp41 virosomal vaccines given by intramuscular priming followed intranasal boosting (26). As such, our passive immunization data provide a blueprint to develop HIV/AIDS vaccines that mobilize mucosal as well as systemic immune defenses.

## Data Availability Statement

The original contributions presented in the study are included in the article/**Supplementary Material**. Further inquiries can be directed to the corresponding author.

## Ethics Statement

The animal study was reviewed and approved by Institutional Animal Care and Use Committee of the University of Louisiana at Lafayette.

## Author Contributions

RR and FV designed the study. JF oversaw animal experiments. SL, HS, DH, and SG performed *in vitro* assays, KR genotyping, and PX post-challenge virus titrations. SG analyzed data. SR performed statistical analyses. AL, EC, and DC contributed the mAbs. SG and RR wrote the report. All authors contributed to the article and approved the submitted version.

## Funding

This work was supported by the National Institutes of Health grant P01 AI048240 to RR.

## Conflict of Interest

AL, DC and EC are employees of Vir Biotechnology Inc. and may hold shares in Vir Biotechnology Inc.

The remaining authors declare that the research was conducted in the absence of any commercial or financial relationships that could be construed as a potential conflict of interest.

## Publisher’s Note

All claims expressed in this article are solely those of the authors and do not necessarily represent those of their affiliated organizations, or those of the publisher, the editors and the reviewers. Any product that may be evaluated in this article, or claim that may be made by its manufacturer, is not guaranteed or endorsed by the publisher.
