# Supplementary material for: Cooperation Between Systemic IgG1 and Mucosal Dimeric IgA2 Monoclonal Anti-HIV Env Antibodies: Passive Immunization Protects Indian Rhesus Macaques Against Mucosal SHIV Challenges
Source: Front Immunol. 2021 Aug 3;12:705592. doi: 10.3389/fimmu.2021.705592 (PMC8370093; doi:10.3389/fimmu.2021.705592)
Supplement: Supplementary file 1 [file DataSheet_1.docx]

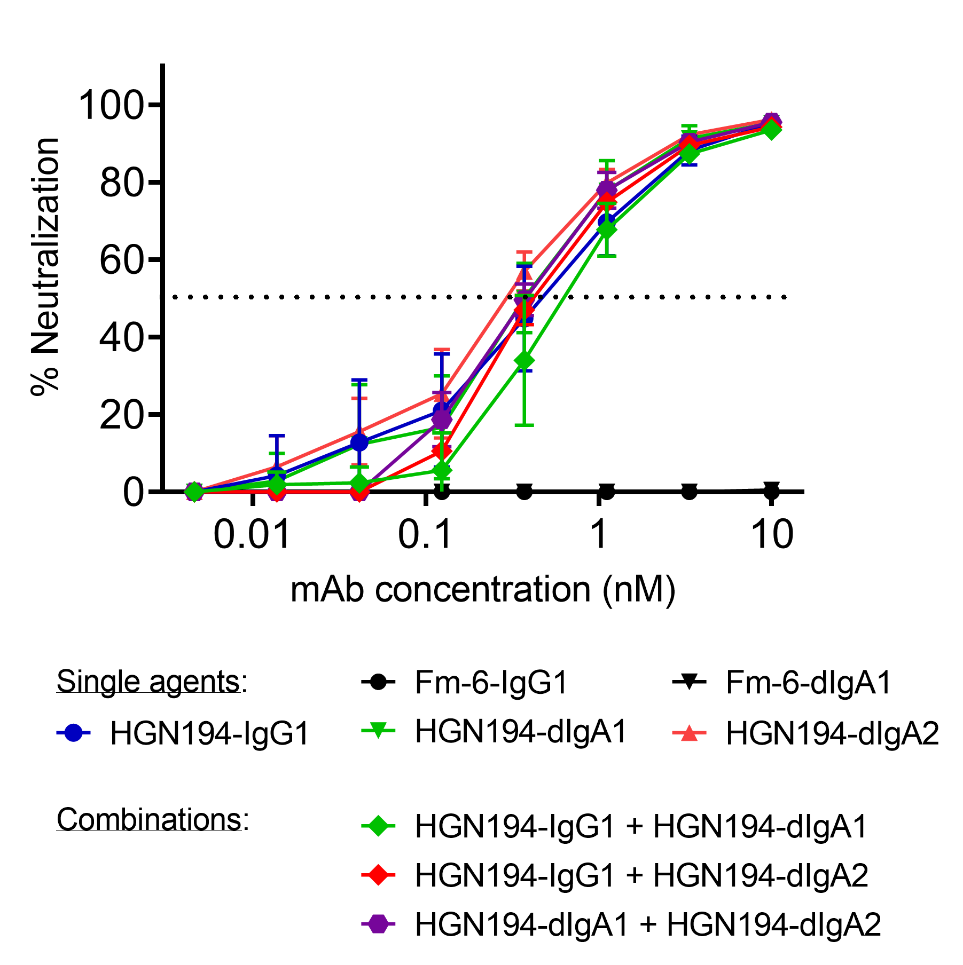


**Supplementary Figure 1.** Neutralization of SHIV-1157ipEL-p by HGN194 mAbs of different classes in TZM-bl assays. The concentration of mAb combinations was the sum of two mAbs mixed at an equal molar ratio. Fm-6-IgG1 or Fm-6-dIgA1, irrelevant negative control mAbs, respectively.
